# Molecular Characterization and Clinical Outcomes of *Klebsiella pneumoniae* in Neonatal Sepsis: A Three-Year Molecular Epidemiological Study

**DOI:** 10.3390/children13091165

**Published:** 2026-08-29

**Authors:** Oscar Villavicencio-Carrisoza, Orly Grobeisen-Duque, Janet Flores-Villanueva, Jimena Lopez-Dueñas, María Guadalupe Martínez-Salazar, Moises Leon-Juarez, Maria Isabel Villegas-Mota, Juan Xicohtencatl-Cortes, Sara A. Ochoa, Irma Eloisa Monroy-Muñoz, Ricardo Figueroa-Damian, Graciela Villeda-Gabriel, Irma Elena Sosa-González, Paola Andrea Santos-Ruiz, Claudia Andrea Cruz-Baquero, Jorge Francisco Cerna-Cortes, Hector Flores-Herrera, Lisbeth Camargo-Marin, Mario Estanislao Guzman-Huerta, Addy Cecilia Helguera-Repetto

**Affiliations:** 1 Departamento de Medicina Traslacional, Instituto Nacional de Perinatología Isidro Espinosa de los Reyes, Mexico City 11000, Mexico; oscar.villavicencio@inper.gob.mx (O.V.-C.); lisbethcamargo@yahoo.com.mx (L.C.-M.); 2 Departamento de Inmunobioquímica, Instituto Nacional de Perinatología Isidro Espinosa de los Reyes, Mexico City 11000, Mexico; orly.grobeisen@gmail.com (O.G.-D.); janet97flores@gmail.com (J.F.-V.); jimenasmd@hotmail.com (J.L.-D.); maria.martinez@inper.edu.mx (M.G.M.-S.); moises.leon@inper.edu.mx (M.L.-J.); hector.flores@inper.gob.mx (H.F.-H.); 3 Escuela Nacional de Ciencias Biológicas, Instituto Politécnico Nacional, Mexico City 11340, Mexico; jorgecerna1008@gmail.com; 4 Secretaría de Salud del Estado de Quintana Roo, Chetumal 77000, Mexico; isabelvillegasmota@gmail.com; 5 Laboratorio de Investigación en Bacteriología Intestinal, Hospital Infantil de México Federico Gómez, Mexico City 11340, Mexico; juanxico@yahoo.com (J.X.-C.); saraariadnah@hotmail.com (S.A.O.); 6 Departamento de Investigación en Salud Reproductiva y Perinatal, Instituto Nacional de Perinatología Isidro Espinosa de los Reyes, Mexico City 11000, Mexico; irmae4901@gmail.com; 7 Departamento de Infectología e Inmunología, Instituto Nacional de Perinatología Isidro Espinosa de los Reyes, Mexico City 11000, Mexico; ricardo.figueroa@inper.gob.mx (R.F.-D.); gracielavilleda@yahoo.com.mx (G.V.-G.); elenasosa9635@gmail.com (I.E.S.-G.); 8 Facultad de Ciencias de la Salud, Universidad Colegio Mayor de Cundinamarca, Bogota 111711, Colombia; psantos@universidadmayor.edu.co (P.A.S.-R.); candreacruz@universidadmayor.edu.co (C.A.C.-B.)

**Keywords:** *Klebsiella penumoniae*, sepsis, neonatal sepsis, virulence, antimicrobial resistance, multidrug resistance, clonal dissemination

## Abstract

**Highlights:**

**What are the main findings?**

- A progressive increase in multidrug resistance, ESBL production, and virulence gene burden was observed in K. pneumoniae isolates causing neonatal sepsis over three years.
- The co-occurrence of resistance and virulence determinants was particularly evident among ST45 isolates belonging to a predominant PFGE cluster, which showed high-risk molecular profiles and associations with adverse neonatal characteristics.

**What are the implications of the main findings?**

- The emergence of high-risk, potentially hospital-adapted K. pneumoniae clones underscores the importance of integrating molecular surveillance into neonatal infection control strategies.
- Combined assessment of antimicrobial resistance, virulence determinants, and clonal relatedness may improve the early recognition of high-risk strains and support targeted infection prevention and antimicrobial stewardship in NICUs.

**Abstract:**

**Background**: *Klebsiella pneumoniae* is one of the leading causes of healthcare-associated neonatal sepsis, with increasing antibiotic resistance and virulence representing major challenges for neonatal care. This study aimed to characterize the molecular epidemiology of *K. pneumoniae* isolates causing neonatal sepsis at a tertiary referral center in Mexico City and to evaluate the relationship between antibacterial resistance, virulence determinants, clonal dissemination, and neonatal outcomes. **Methods**: A retrospective observational study was conducted including 57 *K. pneumoniae* isolates recovered from 39 neonates with culture-confirmed sepsis between 2021 and 2023. Antibiotic susceptibility testing, ESBL production, multidrug resistance, detection of resistance- and virulence-associated genes, multilocus sequence typing, and clinical outcome analyses were performed. **Results**: A progressive increase in antibiotic resistance and virulence was observed throughout the study period. MDR and ESBL-producing isolates increased from 44.4% and 33.3% in 2021 to 78.9% each in 2023, while isolates with high virulence gene burden increased from 33.3% to 73.7%. The most prevalent resistance genes were *blaCTX-M*, *aac(6′)-Ib*, and *aac(3)-IIa*, whereas *uge*, *ycfM*, *fimD,* and *entB* were the predominant virulence determinants. High-virulence gene burden was associated with increased prevalence of *blaCTX-M* and *aac(6′)-Ib*, while ESBL-producing isolates were associated with *ybtS*, and MDR isolates were associated with *ybtS* and *uge*. PFGE identified a predominant clone (Cluster B) with 100% similarity, characterized by ST45 isolates and significantly higher resistance and virulence profiles. High-virulence isolates were associated with thrombocytopenia and extreme prematurity. **Conclusions**: *K. pneumoniae* isolates causing sepsis are increasing antibiotic resistance, virulence gene burden, and clonal dissemination over the three-year study period. The convergence of resistance and virulence determinants highlights the emergence of high-risk hospital-adapted clones and underscores the importance of continuous molecular surveillance to improve infection control and optimize neonatal management.

## 1. Introduction

The Third International Consensus Definition for and Septic Shock (Sepsis-3) defines sepsis as “a life-threatening organ dysfunction caused by a dysregulated host response to infection” [1]. This condition results from an exaggerated inflammatory response characterized by immune dysregulation, excessive cytokine release, endothelial dysfunction, and tissue injury, ultimately leading to septic shock, multiple organ dysfunction, and death if not promptly recognized and treated. Despite advances in critical care and antimicrobial therapy, sepsis remains one of the leading causes of morbidity and mortality worldwide [2,3].

The neonatal period represents one of the most vulnerable stages of life for the development of sepsis due to the immaturity of both the innate and adaptive immune systems. Prematurity, low birth weight, low Apgar scores, prolonged hospitalization, central venous catheterization, parenteral nutrition, mechanical ventilation, and extended stays in neonatal intensive care units (NICUs) are among the most recognized risk factor predisposing newborns to severe bacterial infections. In addition to host-related risk factors, the increasing prevalence of antibiotic-resistant pathogens has further compromised treatment efficacy, making neonatal sepsis a major public health challenge [4,5].

Recent global estimates indicate that sepsis accounts for approximately 166 million cases and 21.4 million deaths each year, with children under five years of age representing one of the most affected populations [6]. Neonatal sepsis alone has an estimated global incidence of 3930 cases per 100,000 live births, disproportionately affecting low- and middle-income countries, where access to timely diagnosis and appropriate antibiotic therapy remains limited [6]. In Latin America, more than 50% of deaths in children under five occur during the neonatal period, with neonatal sepsis representing one of the leading contributors to neonatal mortality [7]. However, despite its clinical relevance, the true burden of neonatal sepsis in Latin America and Mexico remains difficult to determine due to heterogeneous surveillance systems, limited microbiological characterization, and the lack of standardized epidemiological reporting.

In Mexico, the reported incidence of neonatal sepsis ranges from 4 to 15.4 cases per 1000 live births, exceeding the 1.1–8.6 cases per 1000 live births reported in other Latin American countries [8,9]. This burden is even greater among critically ill neonates requiring intensive care. At the Instituto Nacional de Perinatología (INPer), the incidence of culture-confirmed neonatal sepsis was 4.7 cases per 1000 live births in 2023 [10], whereas among infants admitted to the neonatal intensive care unit (NICU), the incidence reached 112.4 cases per 1000 admissions, highlighting the substantial burden of sepsis in this high-risk population [11].

Among the bacterial pathogens causing neonatal sepsis, *Staphylococcus aureus*, *Acinetobacter* spp., *Streptococcus agalactiae*, *Streptococcus pneumoniae*, *Escherichia coli*, and *Klebsiella pneumoniae* are the most frequently reported. Among these, *K. pneumoniae* has emerged as one of the most clinically relevant nosocomial pathogens because of its remarkable capacity to acquire antibiotic resistance and virulence determinants while persisting in the hospital environment. According to the 2024 World Health Organization (WHO) Bacterial Priority Pathogens List, third-generation cephalosporin-resistant and carbapenem-resistant *K. penumoniae* are classified as critical-priority pathogens, underscoring the urgent need for effective surveillance and infection control strategies [12,13,14]. Furthermore, the widespread dissemination of multidrug-resistant (MDR) and extended-spectrum beta-lactamases (ESBL)-producing *K. penumoniae* has substantially reduced therapeutic options and has been consistently associated with prolonged hospitalization, increased health costs, and higher mortality among neonatal, pediatric, and adult patients with bloodstream infections [15,16].

Beyond antibiotic resistance, the pathogenic success of *K. penumoniae* is also driven by the acquisition of virulence-associated determinants involved in iron acquisition, adhesion, biofilm formation, immune evasion, and capsule biosynthesis. The increasing convergence between antibiotic resistance and virulence determinants has become a growing concern, as strains harboring both characteristics may exhibit enhanced pathogenicity, improved bacterial fitness, and reduced susceptibility to available therapies [17]. Although several studies have independently characterized resistance mechanisms or hypervirulent lineages, few have simultaneously investigated antimicrobial resistance, virulence-associated genes, clonal dissemination, and their relationship with neonatal clinical outcomes, particularly in Latin American populations.

Therefore, the aim of this study was to characterize the antibiotic resistance profiles, virulence-associated genes, and clonal relationship of *K. pneumoniae* isolates recovered from neonatal sepsis cases at INPer between 2021 and 2023 and to evaluate their associations with neonatal clinical outcomes. By integrating molecular epidemiology with clinical characteristics, this study seeks to provide evidence to support improved surveillance strategies, optimized antimicrobial use, and strengthen infection prevention and control measures in neonatal intensive care settings.

## 2. Materials and Methods

### 2.1. Study Approval and Data Collection

This study was approved by the Research Ethics Committee of the Instituto Nacional de Perinatología (approval number 212250-3210-11007-04-14). It was designed as a retrospective, cross-sectional, observational study conducted at the Instituto Nacional de Perinatología between January 2021 and December 2023.

The inclusion criteria included neonates with culture-confirmed sepsis caused by *Klebsiella pneumoniae* isolated from blood and cerebrospinal fluid. Newborns with clinical sepsis in whom microorganisms other than *K. pneumoniae* were isolated were excluded. Cases initially diagnosed as *K. pneumoniae* infections but subsequently reclassified as different microorganisms following molecular identification were also excluded from the analysis.

Patient data were collected from electronic clinical records (Expediente Clínico Electrónico), the legally required electronic health record system in Mexico that contains comprehensive patient medical information. All patient information was anonymized before analysis.

The neonatal variables analyzed included fever, hypothermia, tachycardia, bradycardia, tachypnea, bradypnea, apnea, respiratory distress syndrome, leukopenia, leukocytosis, neutrophilia, thrombocytopenia, thrombosis, meningitis, gastroschisis, enterocolitis, intrauterine growth restriction, gestational age, and birth weight. Maternal variables included in this study were cervicovaginitis, urinary tract infection, premature rupture of membranes, chorioamnionitis, diabetes, gestational diabetes, obesity, and preeclampsia.

Gestational age was also classified according to the WHO as follows: extremely preterm (<28 weeks), very preterm (28–32 weeks), preterm (32–37 weeks), and term (>37 weeks) [18].

### 2.2. Biological Material

Clinical isolates were cultured in Tryptic Soy Broth (TSB) (Beckton Dickinson and Company, 07417 Franklin Lakes, NJ, USA) and incubated at 37 °C for 24 h. Subsequently, viable isolates were plated on MacConkey agar (Beckton Dickinson and Company, 07417 Franklin Lakes, NJ, USA) and incubated at 37 °C for 24 h. Each isolate was preserved in TSB supplemented with 15% glycerol (Merk KGaA, 64293 Darmstadt, Germany) at −70 °C.

Genomic DNA was extracted using the Quick-gDNA™ MiniPrep kit (Zymo Research Corp, 92614 Irvine, CA, USA) according to the manufacturer’s instructions.

Strain confirmation and antimicrobial susceptibility testing were performed in the Clinical Microbiology Laboratory using the VITEK 2^®^ system (bioMérieux SA, 69280 Marcy L’Étoile, Lyon, France) with AST-N271 and AST-N272 cards.

Molecular identification of *K. pneumoniae* was performed by real-time PCR targeting the *tonB* gene. Amplification consisted of an initial denaturation at 95 °C for 5 min, followed by 30 cycles of denaturation at 95 °C for 15 s and annealing at 58 °C for 15 s, using the QuantStudio^TM^ 5 Real-Time System with SYBR^TM^ Green I Master Mix (Thermo Fisher Scientific, 02451 Waltham, MA, USA). Species identification was confirmed by melt curve analysis using a ramp rate of 0.05 °C/s, yielding a characteristic melting temperature (Tm) of 91.6 °C.

### 2.3. Molecular Detection of Antibiotic Resistance and Virulence

PCR analysis was performed to detect four beta-lactam resistance genes, three aminoglycoside resistance genes (Table 1), and eight *Klebsiella pneumoniae* virulence-associated genes (Table 2),

For amplification of virulence genes, each reaction contained 100 ng of genomic DNA, 500 nM of each primer, and 0.05 U/μL of Taq polymerase. For resistance genes, reactions contained 100 ng of genomic DNA, 320 nM of each primer, and 0.025 U/μL of Taq polymerase (Thermo Fisher Scientific).

PCR conditions consisted of an initial denaturation at 94 °C for 5 min, followed by 30 cycles for virulence genes and 35 cycles for resistance genes, each including denaturation at 94 °C for 40 s, annealing at gene-specific temperatures shown in Table 1 and Table 2, and extension at 72 °C for 1 min, followed by a final extension at 72 °C for 5 min.

Amplified products were separated by agarose gel electrophoresis, stained with SYBR^TM^ Green, and visualized under UV light using a UVP EpiChemi II Darkroom imaging system (Analytik Jena, Jena, Germany).

### 2.4. Determination of Antimicrobial Resistance Patterns

The antimicrobial susceptibility profiles of *K. pneumoniae* strains were determined according to the 2025 Clinical and Laboratory Standards Institute (CLSI) using the WHONET 2025 platform for data interpretation. Susceptibility was assessed against the following antimicrobial agents: amikacin, ampicillin/sulbactam, cefoxitin, cefepime, ceftriaxone, ceftazidime, ciprofloxacin, piperacillin/tazobactam, doripenem, ertapenem, gentamicin, imipenem, meropenem, and tigecycline.

### 2.5. Molecular Typing of Klebsiella pneumoniae by PFGE

Clinical isolates were genotyped by PFGE to evaluate their clonal relatedness. Briefly, isolates were cultured on blood agar (Becton Dickinson, Franklin Lakes, NJ, USA) and incubated at 37 °C for 24 h. Bacterial cells were subsequently resuspended in PIV buffer (2 M Tris-HCl, pH 7.6; 5 M NaCl; Merk KgaA) and washed three times with the same buffer.

A total of 120 µL of the bacterial suspension was mixed with 2%low-melting-point agarose, 10 μL of proteinase K, and 10 μL of lysozyme. After solidification, agarose plugs were transferred to 1.5 mL microcentrifuge tubes containing lysis buffer (2 M Tris-HCl, pH 7.6; 5 M NaCl; 0.5 M EDTA, pH 7.6 [Merk KGaA]; 5% Brij58; 10% deoxycholate [ThermoFisher Scientific]; 20% sarcosyl [Merk KGaA]; 10 mg/mL lysozyme [Thermo Fisher Scientific]) and incubated at 37 °C for 12 h.

The plugs were subsequently incubated in ESP buffer (0.5 M EDTA, pH 9; 20% sarcosyl; 20 mg/mL proteinase K (Thermo Fisher Scientific) at 50 °C for 24 h. Thereafter, plugs were washed three times with ultrapure water and three additional times with 1X TE buffer at 50 °C for 15 min per wash.

For DNA digestion, plugs were equilibrated in 1X XbaI restriction buffer for 30 min at 37 °C and then incubated with 30 U of XbaI (Thermo Fisher Scientific) for 3 h at 37 °C.

PFGE was performed on 1% agarose gels in a CHEF Mapper^®^ XA system (Bio-Rad Laboratories, Hercules, CA, USA) under the following conditions: 6 V/cm, 120° angle, initial pulse time of 2.2 s, final pulse time of 54.2 s, linear ramp, at 14 °C for 24 h.

Restriction patterns were visualized using the EpiChemi II Darkroom imaging system (Analytik Jena, Jena, Germany) and analyzed with BioNumerics^®^ v7.6 (Applied Maths, BioMérieux, Sint-Martens-Latem, Belgium) using the Dice similarity coefficient and the unweighted pair group with arithmetic mean (UPGMA) with 2% optimization and 4% position tolerance. These parameters enable the accurate assessment of clonal relationships among the isolates [24].

### 2.6. Multilocus Sequence Typing Using Sanger Method

Multilocus sequence typing (MLST) was performed according to the protocol described by Diancourt L et al. and available through the Institut Pasteur PubMLST database [25].

Following PCR amplification, products were purified using the AFTSpin Multifunction DNA purification KIT RK30100 (AB clonal technology 500W Cummings Park, Woburn, MA, USA). Purified amplicons were sequenced using the BigDye Terminator v3.1 Cycle Sequencing Kit (Applied Biosystems, Foster City, CA, USA) according to the manufacturer’s instructions on an ABI PRISM^®^ 3130 Genetic Analyzer (Applied Biosystems, Foster City, CA, USA).

The resulting chromatograms were analyzed using BioEdit v7.2 (Ibis Biosciences, Carlsbad, CA, USA) to ensure sequence quality and alignment accuracy. Allelic profiles were confirmed through comparison with reference sequences using the NCBI BLASTn database v.2.17.0.

### 2.7. Clonality Assessment and Statistical Analysis

Clonality analysis was interpreted according to the criteria proposed by Tenover et al. for outbreak classification. Isolates with 100% similarity were considered indistinguishable, those with 85–90% similarity were classified as closely related, isolates with 70–80% similarity were considered possibly related, and those showing less than 65% similarity were classified as unrelated [26].

For the statistical analysis, four independent variables were evaluated to assess association with molecular and clinical characteristics. The first comparison was based on the presence or absence of extended-spectrum beta-lactamases (ESBL); the second compared multidrug-resistant (MDR) and antimicrobial-susceptible isolates; the third classified isolates according to their virulence gene burden as “low virulence gene burden” (1–4 virulence-associated genes) or “high virulence gene burden” (5–8 virulence-associated genes); and the fourth compared the PFGE clusters identified by dendrogram analysis.

The four analytical domains were defined according to the objectives of the study. Individual associations evaluated within these domains were considered exploratory and hypothesis-generating. In particular, comparisons of individual virulence genes between high- and low-virulence gene-burden groups were intended to describe the molecular composition of these groups rather than to establish independent predictive associations.

For inferential comparisons, only the first isolate recovered from each neonate was considered (39). Because PFGE data were unavailable for two of these isolates, cluster-based statistical analyses were performed on 37 independent isolates. All available isolates with PFGE profiles (*N* = 54) were nevertheless retained for descriptive molecular epidemiological and clonal analyses.

Because none of the isolates fulfilled the currently accepted molecular criteria for hypervirulent *K. pneumoniae* (hvKp), isolates were classified according to their cumulative virulence-associated genes rather than being designated as hypervirulent. Isolates harboring five to eight virulence-associated genes were categorized as high virulence gene burden, whereas those carrying one to four virulence-associated genes were classified as low virulence gene burden. This approach was intended solely to compare the cumulative burden of accessory virulence determinants and should not be interpreted as a molecular definition of hvKp. [27].

Statistical analyses were conducted using IBM SPSS Statistics 27 (IBM, 10504-1722 Armonk, NY, USA). Descriptive statistics included percentages and measures of central tendency. Associations between categorical variables were evaluated using the chi-square test, with statistical significance defined as *p* < 0.05. When the expected frequency in any cell of a 2 × 2 contingency table was less than five, Fisher’s exact test (two-tailed) was applied. Odds ratios (ORs) and 95% confidence intervals (95% CI) were calculated to estimate the strength of associations [9].

Forest plots were generated using the ForestPlotter package version 1.1.1 in the R platform environment V. 4.5.2.

## 3. Results

From January 2021 to December 2023, 39 neonates were diagnosed with sepsis caused by *Klebsiella pneumoniae*. Of these, 9 were term, 9 preterm, 13 very preterm, and 8 extremely preterm. The clinical characteristics of the study cohort are summarized in Supplementary Table S1. Some neonates presented with 2 and 5 *K. pneumoniae* isolates at different time points, resulting in a total of 57 strains, which were distributed as follows: 9 in 2021, 29 in 2022, and 19 in 2023.

A temporal variation in the distribution of resistance and virulence genes was observed between 2021 and 2023 (Table 3). The most prevalent resistance genes among all isolates were *blaCTX-M* (45.6%), *aac(6′)-Ib* (49.1%), and *aac(3)-IIa* (42.1%), all showing a progressive increase over time. Notably, *blaCTX-M* rose from 11.1% in 2021 to 78.9% in 2023, while *aac(6′)-Ib*, absent in 2021, was detected in more than two-thirds of the isolates by 2023. In contrast, *blaTEM* exhibited a decreasing trend during the study period, declining from 55.6% in 2021 to 31.6% in 2023.

Regarding virulence genes, the highest overall frequencies corresponded to *uge* (68.4%), *ycfM* (66.7%), *fimD* (64.9%), and *entB* (59.6%). A marked increase was observed for *uge*, *ybtS*, and *wabG*, which reached their highest frequencies in 2023 (94.7%, 94.7%, and 68.4%, respectively). Conversely, *mrkC* decreased from 77.8% in 2021 to 57.9% in 2023, and *iucA* was only sporadically detected (1.8% of isolates) (Table 3).

Overall, these findings show a progressive rise in the diversity and frequency of genes associated with antimicrobial resistance and virulence, particularly among the most recent isolates, and during the study period.

A progressive increase in the MDR phenotype, extended-spectrum beta-lactamase (ESBL) production, and virulence potential was observed among *K. pneumoniae* isolates between 2021 and 2023 (Table 4). The proportion of MDR strains increased from 44.4% in 2021 to 78.9% in 2023, representing 64.9% of all isolates. Similarly, ESBL-producing strains showed a steady rise, from 33.3% in 2021 to 78.9% in 2023.

Regarding virulence grouping, isolates classified as having high virulence-gene burden also exhibited a gradual increase, from 33.3% in 2021 to 73.7% in 2023, accounting for more than half of the total isolates (54.3%).

Isolates were classified according to MDR status, ESBL production, and virulence-gene burden to explore their associations with molecular and clinical characteristics. MDR and ESBL-producing isolates each represented 64.9% of the total isolates (37/57), whereas 54.3% were classified as having a high virulence-gene burden (Table 4).

Overall, these results show an increment in antimicrobial resistance and virulence-gene burden during the study period, suggesting the increasing circulation of more resistant and potentially pathogenic *K. pneumoniae* strains.

### 3.1. Association with ESBL Production

**Figure 1:**
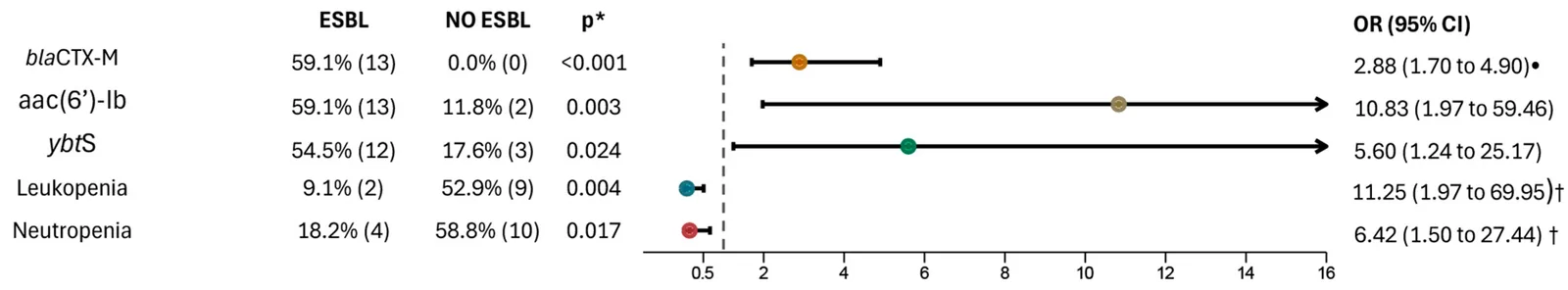
Association of ESBL presence with clinical and molecular variables in *Klebsiella pneumoniae* isolated from neonates diagnosed with sepsis. An association was observed with two genes related to antibiotic resistance, one virulence gene and two clinical variables. The dashed line indicates the reference value (1) on the X-axis, and the horizontal lines crossing it denote lack of a significant association with risk. ESBL: Extended-Spectrum β-Lactamase; NO ESBL: No Extended-Spectrum β-Lactamase; OR: Odds Ratio; CI—Confidence Interval; * *p* < 0.05; † Risk Over NO ESBL Group; ● Relative Risk (RR) was reported because the OR could not be estimated due to a zero cell.

An association analysis based on ESBL status revealed significant relationships with several molecular determinants. The *blaCTX-M*-positive isolates were 2.88 times more likely to exhibit the ESBL phenotype than *blaCTX-M*-negative isolates (*p* < 0.001; RR 2.88, 95% CI 1.70–4.90). Because no *blaCTX-M*-positive isolates were detected in the ESBL-negative group, the odds ratio could not be estimated and the relative risk was reported instead. Overall, 59.1% of ESBL-producing isolates carried *blaCTX-M*, supporting a strong association between this determinant and the ESBL phenotype in our cohort. Given the exploratory nature of the analysis and the limited sample size, this finding should be confirmed in larger studies (Figure 1).

Surprisingly, the *aac(6′)-Ib* gene (*p* 0.003, OR 10.83, 95% CI 1.97–59.46) was present in 59.1% of ESBL-positive isolates; however, its presence is related to resistance to aminoglycosides rather than β-lactams, suggesting that these strains may have a broader resistance profile, increasing the risk of therapeutic failure with aminoglycosides (Figure 1).

Regarding virulence genes, a significant association with ESBL was observed for *ybtS* (*p* 0.024, OR 5.60, 95% CI 1.24–25.17), suggesting that ESBL-positive strains may simultaneously harbor virulence factors that enhance their pathogenic potential (Figure 1).

Neonates infected with non-ESBL-producing strains showed significant associations with leukopenia (*p* = 0.004; OR 11.25, 95% CI 1.97–69.95) and neutropenia (*p* = 0.017; OR 6.42, 95% CI 1.50–27.44). These findings indicate that both hematological alterations were more frequently observed among neonates infected with non-ESBL-producing strains in this cohort. Given the exploratory nature of the analysis and the wide confidence intervals, these associations should be interpreted cautiously (Figure 1).

### 3.2. Association with Multidrug Resistance Profile

**Figure 2:**
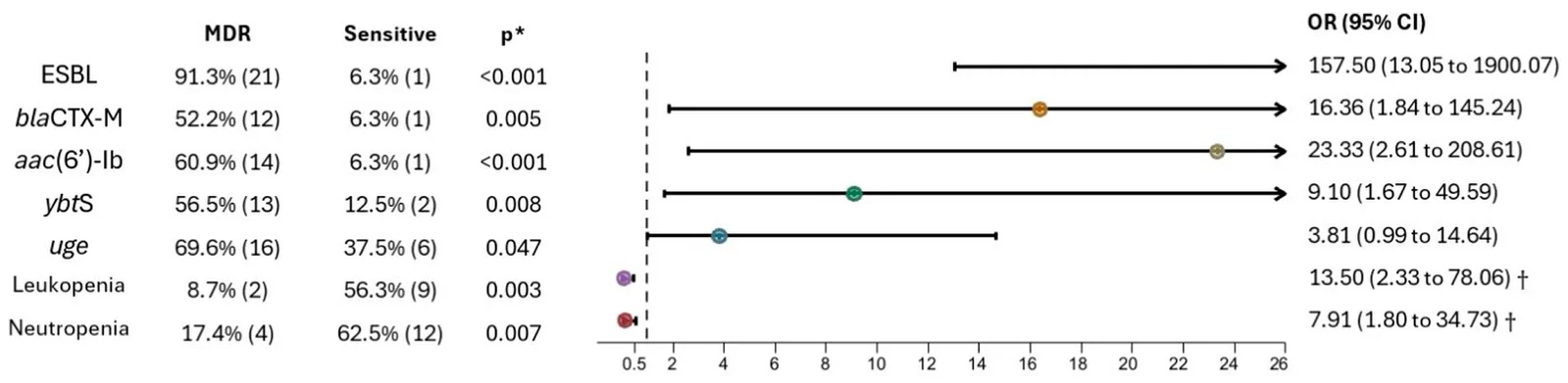
Association of multidrug resistance profiles with clinical and molecular variables in *Klebsiella pneumoniae* isolated from neonates diagnosed with sepsis. An association analysis was performed between multidrug-resistant (MDR) strains and different clinical and molecular variables. The resistance profile was determined using the free WHO-Net platform. The dashed line indicates the reference value (1) on the X-axis, and the horizontal lines crossing it represent lack of a significant association with risk. MDR: Multidrug-Resistant; OR: Odds Ratio; CI: Confidence Interval; * *p* < 0.05; † Risk Over Sensitive Group.

When comparing the multidrug resistance profile with different strain features and clinical outcomes, we observed, as expected, significant associations with the MDR phenotype and resistance-associated genes, specifically for *blaCTX-M* (*p* 0.005; OR = 16.36; 95% CI 1.84–145.24) and *aac(6′)-Ib* (*p* < 0.001, OR 23.33, 95% CI 2.61–208.61) (Figure 2).

Furthermore, the presence of extended-spectrum beta-lactamase (ESBL) was also strongly associated with the MDR phenotype (*p* < 0.001, OR 157.50, 95% CI 13.05–1900.07), suggesting that MDR isolates were highly likely to exhibit an ESBL phenotype (Figure 2).

Regarding virulence genes, *ybtS* (*p* 0.008, OR 9.10, 95% CI 1.67–49.59) was significantly associated with MDR strains and *uge* (*p* 0.047, OR 3.81, 95% CI 0.99–14.64) showed a borderline association (*p* = 0.047; OR 3.81, 95% CI 0.99–14.64), which should be interpreted cautiously given the wide confidence interval and the inclusion of the null value; nevertheless, these results suggest that these resistant strains may simultaneously harbor virulence factors that enhance their pathogenic potential (Figure 2).

Regarding neonatal outcomes, a significant association was found between susceptible strains and the occurrence of leukopenia (*p* 0.003, OR 13.50, 95% CI 2.33–78.06), as well as neutropenia (*p* 0.007, OR 7.91, 95% CI 1.80–34.73). These findings indicate that both hematological alterations were more frequently observed among neonates infected with susceptible strains in this cohort. (Figure 2).

### 3.3. Association with Virulence-Gene Burden Profiles

When comparing virulence-gene burden and resistance determinants, only two of the seven resistance genes evaluated were significantly enriched among isolates with a high virulence-gene burden: *blaCTX-M* (*p* = 0.050) and *aac(6′)-Ib* (*p* = 0.019). These findings indicate a higher likelihood of detecting *bla*CTX-M (81.9%) and *aac*(6′)-Ib (86.9%) in *K. pneumoniae* strains harboring more than five virulence-associated genes (high virulence-gene burden strains) (Table 5).

Also, isolates classified as having high virulence-gene burden demonstrated a significantly higher prevalence of key virulence determinants. This group was characterized by cumulative carriage of *fimD* (*p* 0.015), *mrkC* (*p* 0.010), *entB* (*p* < 0.001), *uge* (*p* < 0.001), *wabG* (*p* < 0.001), *ybtS* (*p* 0.019) and *ycfM* (*p* 0.002), which together might facilitate critical pathogenic processes such as biofilm formation, iron acquisition, and evasion of the neonatal immune system (Table 5).

**Figure 3:**
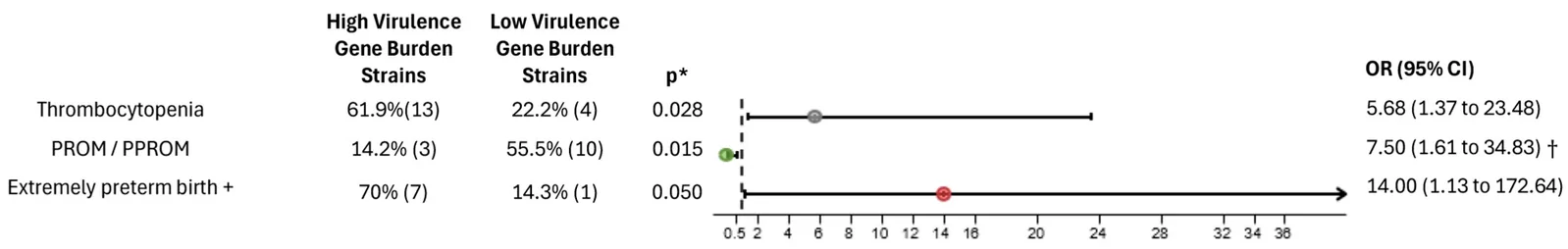
Characteristics associated with virulence-gene burden. High-virulence-gene burden strains were defined as those carrying 5 to 8 virulence genes, whereas low-virulence-gene burden strains carried 1 to 4 genes. The dashed line indicates the reference value (1) on the X-axis, and the horizontal lines crossing it represent a lack of a significant association with risk. OR: Odds Ratio; CI—Confidence Interval; PROM—Premature Rupture of Membranes; * *p* < 0.05; + Extremely Preterm Birth vs. Preterm Birth; † Risk Over Low Virulence Strains Group.

Furthermore, regarding clinical outcomes, strains classified as high virulence-gene burden showed an association with thrombocytopenia (*p* 0.028, OR 5.68, 95% CI: 1.37–23.48) and were more frequently identified in extremely preterm neonates (7 neonates versus 1 with a low virulence-gene burden strain) (*p* 0.05, OR 14.00, 95% CI: 1.13–172.64). Additionally, neonates born after PROM (premature rupture of membranes) and PPROM (preterm premature rupture of membranes) showed a higher frequency of infection with low virulence-gene burden strains, with 55.5% vs. 14.2% in those with high virulence-gene burden strains (*p* 0.006, OR 7.50, 95% CI: 1.61–34.83) (Figure 3).

### 3.4. Clonal Analysis Profile

**Figure 4:**
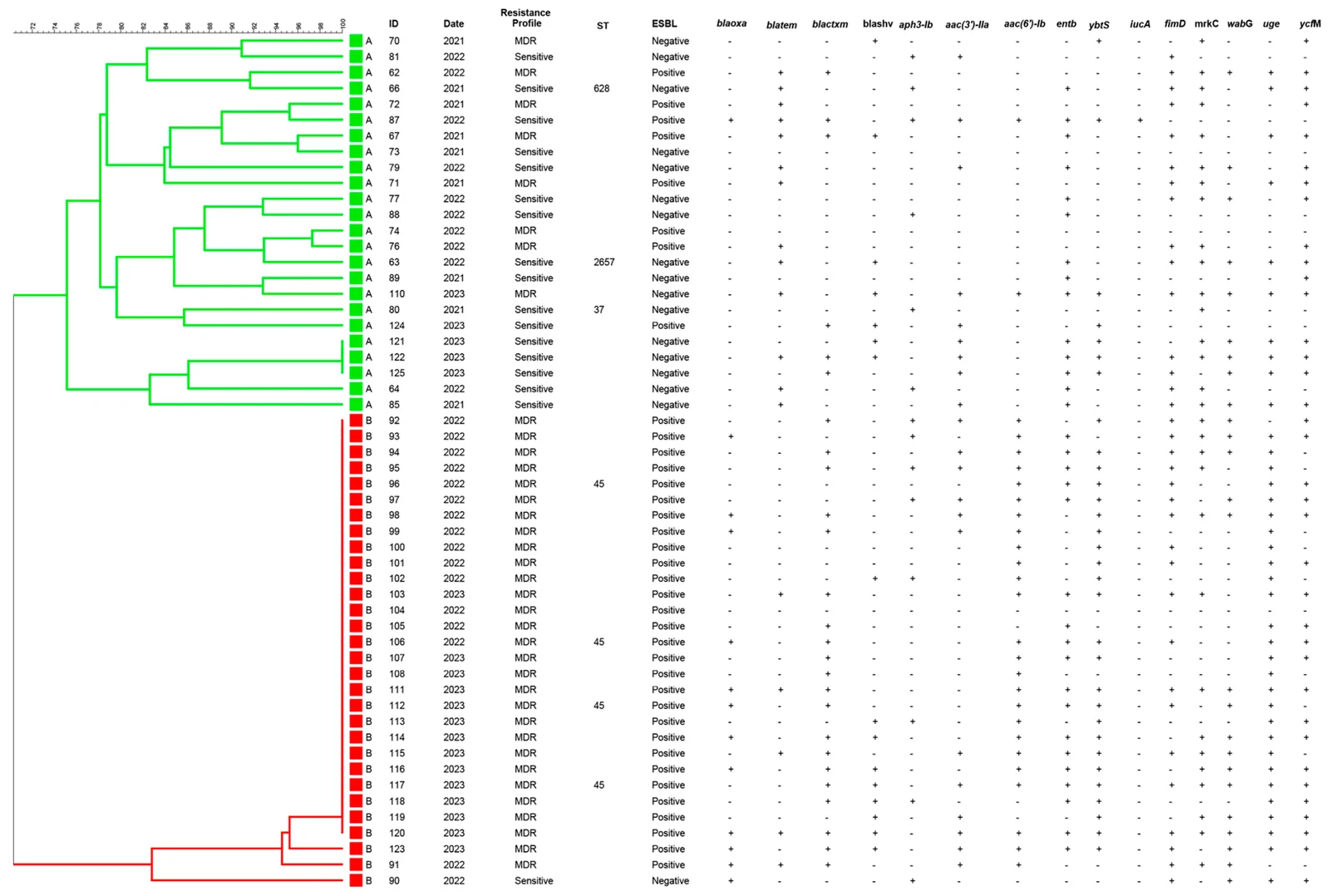
Clonality evaluation of *Klebsiella pneumoniae* strains. A total of 54 strains were analyzed by PFGE and grouped into two related clusters, designated A (24 strains) and B (30 strains), corresponding to the periods 2021–2023 and 2022–2023, respectively. In cluster A, only 8 strains exhibited a multidrug-resistant (MDR) profile. In contrast, in cluster B, 29 strains showed an MDR profile, all of them positive for ESBL production. Additionally, it shows the MLST profiles and the presence or absence of the different genes identified in this study.

Clonal analysis revealed that 24 strains clustered in cluster A, showing more than 75% similarity. In contrast, in cluster B, 27 of 30 strains exhibited 100% similarity, suggesting the circulation of the same clone in the hospital during the 2022–2023 period (Figure 4).

In the right panel of Figure 4, the sequence types (ST) are shown, with four different types identified: ST628, ST2657, ST37, and ST45. Additionally, the presence (+) or absence (-) patterns of the resistance and virulence genes included in this study are displayed, allowing the identification of potential associations between these genes and the analyzed isolates (Figure 4).

### 3.5. Relationship Between Clusters and Molecular and Clinical Variables

Significant differences were observed between clusters A and B regarding resistance and virulence profiles. In cluster B, 93.8% of the strains were both ESBL-producing (*p* < 0.001, OR 30.00, 95% CI 3.26–275.74) and multidrug-resistant (MDR) (*p* < 0.001, OR 24.37, 95% CI 2.68–221.65), indicating that most strains in this cluster exhibit a highly resistant profile.

**Figure 5:**
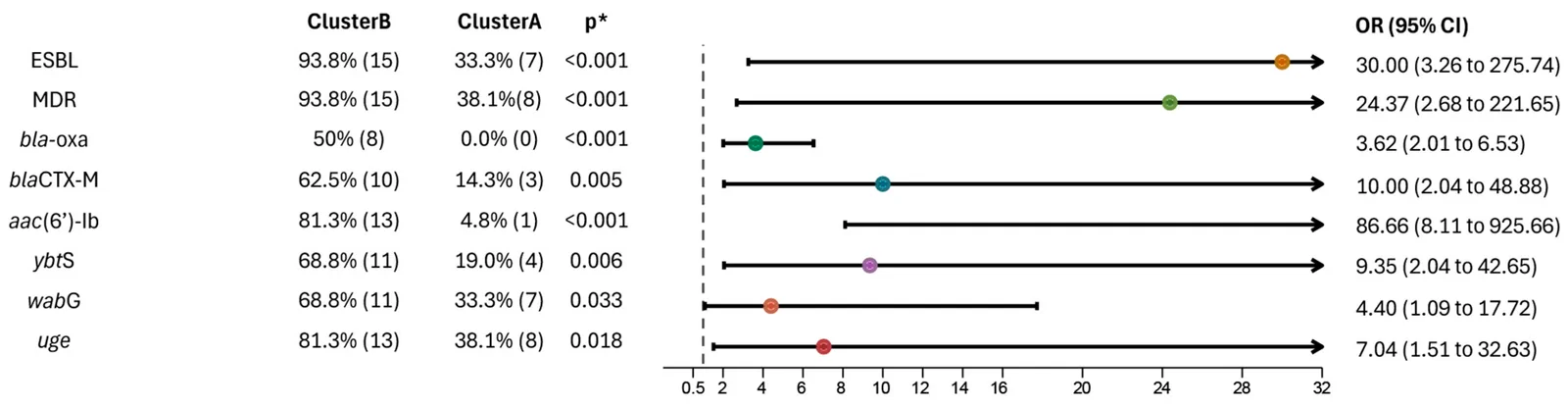
Association of clusters with molecular variables of *Klebsiella pneumoniae* in neonates diagnosed with sepsis. Cluster B exhibited a predominantly multidrug-resistant and ESBL-producing phenotype, in which three antibiotic resistance genes and three virulence genes were identified. The dashed line on the X-axis represents the reference value of 1; the horizontal lines crossing it indicate the absence of a significant association with risk. ESBL: Extended-spectrum β-lactamase; MDR: Multi-Drug Resistance; OR: odds ratio; * *p* < 0.05.

The frequency of the genes *blaOXA* (*p* < 0.001, OR 3.62, 95% CI 2.01–6.53), *blaCTX-M* (*p* 0.005, OR 10.00, 95% CI 2.04–48.88), and *aac(6′)-Ib* (*p* < 0.001; OR 86.66; 95% CI 8.11–925.66) was also significantly higher in cluster B (Figure 5).

Regarding virulence genes, *ybtS* (*p* 0.006; OR = 9.35; 95% CI 2.04–42.65), *wabG* (*p* 0.033; OR = 4.40; 95% CI 1.09–17.72) and *uge* (*p* 0.018; OR = 7.04; 95% CI 1.51–32.63) were significantly more frequent in cluster B. These findings indicate that cluster B was characterized by a higher prevalence of virulence-associated determinants, together with the predominantly resistant phenotype described above (Figure 5). No significant associations were identified between PFGE clusters and clinical outcomes among neonates with sepsis.

## 4. Discussion

This study provides an up-to-date overview of the molecular and clinical characteristics of *K. pneumoniae* strains associated with neonatal sepsis between 2021 and 2023, demonstrating a progressive increase in antimicrobial resistance, virulence-associated genes and clonal dissemination at INPer. These findings reflect a concerning pattern during the study period toward increasingly resistant strains with enhanced prevalence of virulence-associated determinants, representing a significant challenge for neonatal care.

Several studies have documented a global increase in antibiotic resistance among *K. pneumoniae* isolates in recent years. Özkavaklı et al. reported that *K. pneumoniae* was the most frequently isolated Gram-negative pathogen associated with late-onset sepsis between 2015 and 2022, with increasing resistance to cephalosporins and aminoglycosides; however, no resistance to meropenem was detected during the most recent study period (2021–2022) [28]. In contrast, the multicenter NeoOBS study by Russell et al. demonstrated a high prevalence of carbapenem resistance among neonatal pathogens, raising concerns regarding the effectiveness of WHO-recommended antibiotic regimens for neonatal sepsis [29]. Similarly, data from South Africa showed a shift from ESBL-producing strains toward increasing carbapenem resistance over a 10-year period [30]. Other reports from Egypt, India, and additional regions have described rising rates of both ESBL and carbapenem resistance, along with increased resistance to cephalosporines and aminoglycosides, trends that are consistent with our findings [31,32,33].

A similar pattern was observed in our cohort during the study period. By 2023, the frequencies of both MDR and ESBL-producing isolates had reached 78.9% (Table 4). Genotypic analysis also showed an increased frequency of several resistance determinants, particularly *blaCTX-M* and *blaSHV*, associated with β-lactam resistance, as well as *aac(3)-IIa* and *aac(6′)-Ib*, which are associated with aminoglycoside resistance.

Beyond antibiotic resistance, an increased virulence-gene burden has also been reported worldwide. In our cohort, the proportion of isolates classified as high virulence-gene burden increased from 33.3% in 2021 to 73.7% in 2023. A similar global tendency was described by Lam et al., who analyzed over 9000 genomes and demonstrated a progressive increase in virulence score over time. In their study, higher virulence scores were largely associated with siderophore systems such as aerobactin (*iuc*) and yersiniabactin (*ybt*). In our cohort, although classical hypervirulence markers were not systematically predominant, we observed a marked increase in frequency of genes such as *uge*, *ybtS*, and *wabG*, reaching frequencies of 94.7%, 94.7%, and 68.4% in 2023, respectively [17].

Importantly, Lam et al. also described the phenomenon of convergence, in which strains exhibit both elevated virulence scores and multidrug resistance, particularly ESBL production or carbapenem resistance. This convergence has been associated with the acquisition of virulence plasmids by MDR clones or, conversely, the acquisition of resistance determinants by hypervirulent lineages [17]. Although our study did not investigate the genomic context or mechanisms of gene acquisition, the concurrent increase in virulence-gene burden and antimicrobial resistance observed in our cohort may suggest a similar convergence pattern. Plasmid-mediated horizontal gene transfer represents a plausible mechanism that could contribute to this pattern; however, this hypothesis requires confirmation in future genomic studies.

To characterize the virulence-associated genetic profile of the isolates, we evaluated the presence of selected virulence-associated genes previously described in *K. pneumoniae*. These genes were chosen because they represent different functional categories involved in bacterial pathogenicity. Adhesion-related genes (*fimD* and *mrkC*) were included due to their reported role in attachment and biofilm formation. Genes associated with outer membrane structure and host interaction, such as *ycfM*, were also assessed. Additionally, *wabG* and *uge*, which are involved in lipopolysaccharide and capsular polysaccharide biosynthesis, were analyzed given their contribution to structural components linked to immune interaction. Furthermore, iron acquisition systems were represented by the siderophore-associated genes *entB*, *ybtS*, and *iucA*, which have been widely described in clinical isolates of *K. pneumoniae* [19,20,21,22,23]. The detection of these genes allowed us to construct a virulence-gene burden profile for each isolate, which was subsequently used to categorize strains for comparative analysis.

Isolates were categorized as high- and low-virulence-gene burden groups based on the number of detected virulence-associated genes. Within the high-burden group, we observed an enrichment of virulence determinants including *entB*, *fimD*, *mrkC*, *wabG*, *uge*, and *ycfM.* These genes are implicated in iron acquisition, adhesion, biofilm formation, and capsule-associated functions, all of which contribute to bacterial virulence potential and host interaction. While these determinants are considered accessory rather than defining markers of virulence, their cumulative presence may enhance adaptive and survival capacity. Notably, Mukherjee et al. reported that hypervirulent strains harbored accessory genes such as *wabG*, *fimH*, *entB*, *uge*, and *ybtS* at higher frequencies compared to classical strains [34]. Although our isolates were not classified as hypervirulent, the partial overlap in virulence-gene profiles suggests that classical strains may progressively accumulate virulence determinants. While this enrichment does not indicate true hypervirulence, it may reflect an ongoing shift that might enhance pathogenic potential within classical *K. penumoniae* populations.

In our study, a higher virulence-gene burden was associated with increased prevalence of *blaCTX-M* and *aac (6’)-Ib*, suggesting a potential link between enhanced pathogenic potential and antimicrobial resistance. Shrestha et al. similarly reported that an increased number of accessory virulence factors was associated with MDR status, particularly in strains harboring beta-lactamase genes, siderophores, and aminoglycoside resistance determinants [35]. Furthermore, this convergence of virulence and resistance determinants has been previously described where siderophores, adhesion factors, and beta-lactamases coexist within the same strain, representing an emerging clinical and epidemiological threat in hospital settings [18].

Antibiotic resistance in *K. pneumoniae* represents a major global health concern. In our cohort, a statistically significant association was observed between MDR and ESBL production, with MDR isolates frequently exhibiting an ESBL phenotype. In both groups, the most prevalent resistance determinant was *blaCTX-M*, a gene family responsible for resistance to third-generation cephalosporins. A large genomics analysis conducted by Sands et al. investigating neonatal sepsis isolates across multiple low- and middle-income countries reported similar patterns, identifying *K. pneumoniae* as one of the leading causes of neonatal sepsis and documenting high levels of MDR worldwide. In that study, approximately 95% of isolates were resistant to ampicillin and 80% to cephalosporines, with *blaCTX-M-15* identified as the most prevalent resistance determinant in nearly 85% of *K. pneumoniae* isolates. The authors also reported a high prevalence of carbapenemase genes such as *blaNDM* and variants of *blaOXA-48* [36].

Furthermore, our MDR isolates showed a higher prevalence of *aac(6’)-Ib*, a gene encoding an aminoglycoside-modifying enzyme, which may further limit therapeutic options. The coexistence of β-lactam and aminoglycoside resistance determinants highlights the increasing complexity of antimicrobial resistance profiles in clinical *K pneumoniae* isolates.

Additionally, MDR and ESBL-producing isolates in our cohort were significantly associated with the presence of two specific virulence determinants, *ybtS* and *uge*. The *uge* gene, involved in capsular biosynthesis and bacterial virulence, has previously been reported in carbapenem-resistant *K. pneumoniae* isolates from adult intensive care unit patients, where its presence was associated with more severe infection profiles [37]. To our knowledge, similar observations have been less frequently described in neonatal populations, highlighting the need for further investigation in this vulnerable group. In addition, Lam et al. have shown that *ybt* represents one of the most prevalent virulence determinants in *K. pneumoniae*, being detected in approximately 40% of MDR isolates worldwide. This study also reported a notable convergence between isolates harboring yersiniabactin-associated loci and those exhibiting high levels of antimicrobial resistance, including ESBL and carbapenem resistance [17].

Given that all infections in out cohort corresponded to late-onset sepsis acquired within the hospital setting, we further explored the genetic relatedness among isolates. Two distinct clusters were identified. Notably, cluster B exhibited 100% similarity, consistent with a shared lineage and suggesting possible intrahospital transmission of a persistent strain.

Cluster B was characterized by a higher resistance and virulence-gene burden compared to cluster A. A large proportion (93.8%) of the isolates within this cluster were MDR and ESBL producers, with increased prevalence of *blaOXA*, *blaCTX-M*, and *aac(6’)-Ib*. In contrast, only one-third of the isolates in cluster A exhibited MDR and ESBL phenotypes. Additionally, four isolates within cluster B belonged to sequence type ST45, a lineage previously described as a high-risk clone associated with antimicrobial resistance and neonatal infection in Europe [17].

Genomic studies have also highlighted that *K. pneumoniae* lineages showing convergence between antimicrobial resistance and virulence-associated determinants may be linked to greater clinical severity and more limited therapeutic options [17]. Consistent with these observations, cluster B isolates in our study exhibited a higher prevalence of virulence-associated genes, including *ybtS*, *wabG*, and *uge*, indicating an enrichment of virulence-associated determinants within this predominantly resistant cluster. The yersiniabactin locus (*ybt*) has been reported as one of the most prevalent virulence determinants among MDR *K. pneumoniae* isolates worldwide, being detected in up to 40% of cases [17]. Moreover, yersiniabactin carriage has been associated with unfavorable clinical outcomes. Sands et al. reported an association between its presence and increased mortality, as well as an earlier onset of sepsis [36].

In contrast, cluster A was composed of more diverse sequence types, including ST628, ST2657, and ST37. ST37 has been identified as a prevalent lineage in low- and middle-income countries, although it is generally associated with lower resistance profiles [36]. Consistent with this pattern, cluster A isolates in our cohort showed a lower prevalence of resistance-associated determinants than those belonging to cluster B.

Regarding neonatal clinical outcomes, we observed that patients infected with non-ESBL-producing and antibiotic-susceptible isolates had a higher frequency of leukopenia and neutropenia. Leukopenia has been consistently described as a common hematological finding in neonatal sepsis and has been associated with increased mortality, particularly in cases caused by *K. pneumoniae* [38,39]. Similarly, Manandhar et al. reported that leukopenia was associated with increased odds of sepsis in neonatal populations, where a high proportion of isolates were MDR [40].

In contrast, there is limited evidence directly correlating neutropenia with infections caused by non-ESBL or antibiotic-susceptible *K. pneumoniae* isolates. Some studies, such as that by Al Benwan et al., have reported increased neutrophil counts in bacteremia cases involving ESBL-producing *K. pneumoniae* and *Escherichia coli*, suggesting that host response patterns may vary depending on bacterial resistance profiles [41]. In our cohort, leukopenia and neutropenia were more frequently observed among neonates infected with antibiotic-susceptible strains. These findings may suggest that even antibiotic-susceptible strains can exhibit sufficient virulence to significantly impact neonatal clinical stability; however, these exploratory associations should be interpreted cautiously, as they may reflect differences in host response or other unmeasured clinical factors rather than a direct effect of the bacterial resistance phenotype.

Additionally, our patients infected with isolates categorized as having a high virulence-gene burden showed a higher incidence of thrombocytopenia. Although no studies have directly evaluated the relationship between virulence-gene burden and thrombocytopenia, this hematological finding has been widely described as a marker of neonatal sepsis and is associated with increased mortality [38,42]. Platelets play an active role in the inflammatory response, and during sepsis, dysregulated immune activation and increased thrombopoietin levels may contribute to bone marrow exhaustion and reduced platelet production. Consequently, thrombocytopenia is considered a well-established marker associated with disease severity in neonatal sepsis [42].

Furthermore, patients infected with isolates exhibiting a high virulence-gene burden were more frequently associated with extreme prematurity. Extreme preterm neonates represent a highly vulnerable population with increased susceptibility to late-onset sepsis. Trong et al. reported a higher incidence of late-onset sepsis in extremely preterm infants, with *K. pneumoniae* identified as the predominant pathogen and associated with more severe clinical presentations [43]. However, this association should be interpreted cautiously, as neonatal characteristics such as gestational age and birth weight were not controlled for in multivariable analyses.

In contrast, in our cohort, infections caused by isolates with a lower virulence-gene burden were more frequently associated with maternal factors such as PROM and PPROM. Multiple studies have demonstrated an increased risk of neonatal infection in the presence of PROM, and Kumar et al. reported a higher risk of early-onset sepsis in neonates born to mothers with PROM, with *K. pneumoniae* being one of the most common causative agents [44,45].

Additionally, Kwon et al. identified PPROM in the context of maternal vaginal colonization as an independent risk factor for early-onset sepsis. However, no studies to date have established a direct relationship between virulence-gene burden in *K. pneumoniae* and maternal risk factors such as PROM or PPROM, underscoring the need for further research in this area [46].

It is important to mention that the observed associations between bacterial characteristics and neonatal outcomes may be influenced by underlying host vulnerability, particularly gestational age and birth weight, and should not be interpreted as independent or causal effects.

This study has several limitations that should be considered when interpreting our findings. First, this was a retrospective, single-center study with a relatively small sample size, which may limit the generalizability of the results; therefore, future multicenter studies including external validation cohorts will be essential to confirm the reproducibility and generalizability of our findings. Owing to the limited sample size, multivariable regression analyses were not statistically appropriate because of the risk of model overfitting. Consequently, all associations were explored using univariate analyses; therefore, isolated statistically significant associations, particularly those with wide confidence intervals or borderline significance, should be interpreted cautiously and considered hypothesis-generating rather than confirmatory. The limited sample size may increase the probability of type I error and reduce the precision of individual effect estimates. Likewise, although an increase in antibiotic resistance and virulence gene burden was observed during the three-year study period, the duration of surveillance does not allow definitive conclusions regarding long-term epidemiological trends. Despite these limitations, this study provides one of the first comprehensive molecular epidemiological characterizations of *K. pneumoniae* causing neonatal sepsis in Mexico. By integrating antimicrobial susceptibility profiles, resistance, and virulence determinants, PFGE and MLST analyses, clonal dissemination, and neonatal clinical outcomes, our findings provide valuable insights into the emergence of increasingly resistant and potentially more pathogenic hospital-associated clones. These results support the implementation of continuous molecular surveillance and may contribute to improving infection prevention strategies and antibiotic management in neonatal intensive care units.

## 5. Conclusions

This study provides a comprehensive molecular and clinical characterization of *K. pneumoniae* isolates causing neonatal sepsis at a tertiary medical center in Mexico between 2021 and 2023. Our findings show an increased frequency of MDR and ESBL-producing isolates, a higher virulence-gene burden, and evidence of clonal dissemination during the study period. Together, these observations suggest the circulation of *K. pneumoniae* isolates combining antimicrobial resistance with a greater burden of virulence-associated determinants in the neonatal intensive care setting.

Importantly, isolates with a higher virulence-gene burden were more frequently associated with antibiotic resistance determinants, consistent with the convergence between resistance and virulence-associated traits reported worldwide. In addition, several molecular characteristics were associated with clinically relevant neonatal features, including thrombocytopenia, extreme prematurity, and hematological alterations. Although these findings may indicate a relationship between bacterial molecular profiles and clinical presentation, they should be interpreted cautiously given the observational design, limited sample size, and potential influence of host-related and clinical confounding factors. These exploratory associations suggest that the clinical relevance of *K. pneumoniae* in neonatal sepsis may extend beyond antimicrobial susceptibility alone and may also involve the distribution of virulence-associated determinants.

The identification of clonal dissemination, particularly involving ST45 isolates characterized by high antimicrobial resistance and high virulence-gene burden, suggests possible nosocomial circulation of these high-risk lineages within the NICU. These findings support the importance of continuous molecular and genomic surveillance, appropriate antimicrobial use, and strengthened infection prevention and control measures to monitor and limit the dissemination of high-risk *K. pneumoniae* clones.

Overall, this study expands the current understanding of the molecular epidemiology of neonatal *K. pneumoniae* infections in Latin America and provides evidence supporting the integration of antimicrobial resistance profiling, virulence-associated determinants, and genomic surveillance into clinical and epidemiological practice to improve neonatal outcomes and guide future prevention strategies.

## Figures and Tables

**Table 1:** Characteristics of *Klebsiella pneumoniae* Antibiotic Resistance Genes Evaluated by PCR.

Gene	Function or Product	Primer Sequence (5′-3′)	Amplicon Size	PCR Annealing	Reference
*blaTEM*	Carbapenemases	F: AAACGCTGGTGAAAGTA R: AGCGATCTGTCTAT	822 bp	48 °C/1 min	[19]
*blaSHV*	Extended-spectrum β-lactamases (ESBLs)	F: ATGCGTTATATTCGCCTGTG R: TGCTTTGTTATTCGGGCCAA	753 bp	50 °C/1 min	[19]
*blaOXA*	Carbapenemases	F: ATATCTCTACTGTTGCATCTCC R: AAACCCTTCAAACCATCC	619 bp	50 °C/1 min	[19]
*blaCTX-M*	Extended-spectrum β-lactamases (ESBLs)	F: CGCTTTGCGATGTGCAG R: ACCGCGATATCGTTGGT	550 bp	54 °C/1 min	[19]
*aac(3′)*-IIa	Aminoglycoside acetyltransferase enzyme	F: CGGAAGGCAATAACGGAG R: TCGAACAGGTAGCACTGAG	700 bp	54 °C/1 min	[20]
*aac(6′)*-Ib	Aminoglycoside acetyltransferase enzyme	F: CATGACCTTGCGATGCTCTA R: GACTCTTCCGCCATCGCTCT	490 bp	62 °C/1 min	[21]
*aph(3″)-Ib*	Aminoglycoside acetyltransferase enzyme	F: GTGGCTTGCCCCGAGGTCATCA R: CCAAGTCAGAGGGTCCAATC	612 bp	58 °C/1 min	[22]

**Table 2:** Characteristics of *Klebsiella pneumoniae* Virulence Genes Evaluated by PCR.

Gene	Function or Product	Primer Sequence (5′-3′)	Amplicon Size	PCR Annealing	Reference
*entB*	Enterobactin synthase	F: ATTTCCTCAACTTCTGGGGC R: AGCATCGGTGGCGGTGGTCA	371 bp	60 °C/30 s	[23]
*fimD*	Type 1 fimbriae	F: GTTACGCCTATCTGAATCTACAGAG R: GACCAGTTGATATCGTCCACG	1114 bp	57 °C/45 s	[23]
*mrkC*	Type 3 fimbriae	F: GCTCAACTCCATCGTCAGC R: CGCGAGTTATTGAACGAGGTG	1056 bp	57 °C/45 s	[23]
*wabG*	Endotoxin	R: CGGACTGGCAGATCCATATC F: ACCATCGGCCATTTGATAGA	683 bp	58 °C/45 s	[23]
*ybtS*	Yersiniabactin	F: GACGGAAACAGCACGGTAAA R: GAGCATAATAAGGCGAAAGA	242 bp	55 °C/30 s	[23]
*ycfM*	Adhesin	F: ATCAGCAGTCGGGTCAGC R: CTTCTCCAGCATTCAGCG	160 bp	50 °C/1 min	[23]
*uge*	Endotoxin	R: GATCATCCGGTCTCCCTGTA F: TCTTCACGCCTTCCTTCACT	535 bp	58 °C/30 s	[23]
*iucA*	Aerobactin siderophore synthase	F: AATCAATGGCTATTCCCGCTG R: CGCTTCACTTCTTTCACTGACAGG	239 bp	58 °C/30 s	[23]

**Table 3:** Temporal distribution of resistance and virulence genes in *Klebsiella pneumoniae* isolates.

Resistance Genes	2021 *n* = 9	2022 *n* = 29	2023 *n* = 19	Total (*n* = 57)
*blaOXA*	0% (0)	24.1% (7)	31.6% (6)	22.8% (13)
*blaTEM*	55.6% (5)	31% (9)	31.6% (6)	35.1% (20)
*blaCTX-M*	11.1% (1)	34.5% (10)	78.9% (15)	45.6% (26)
*blaSHV*	22.2% (2)	6.9% (2)	63.2% (12)	28.1% (16)
*aph(3′)-Ib*	22.2% (2)	37.9% (11)	10.5% (2)	26.3% (15)
*aac(3)-IIa*	11.1% (1)	44.8% (13)	52.6% (10)	42.1% (24)
*aac(6′)-Ib*	0% (0)	51.7% (15)	68.4% (13)	49.1% (28)
**Virulence Genes**				
*entB*	44.4% (4)	51.7% (15)	78.9% (15)	59.6% (34)
*ybtS*	11.1% (1)	41.4% (12)	94.7% (18)	54.4% (31)
*iucA*	0% (0)	3.4% (1)	0% (0)	1.8% (1)
*fimD*	55.6% (5)	75.9% (22)	52.6% (10)	64.9% (37)
*mrkC*	77.8% (7)	48.3% (14)	57.9% (11)	56.1% (32)
*wabG*	11.1% (1)	44.8% (13)	68.4% (13)	47.4% (27)
*uge*	44.4% (4)	58.6% (17)	94.7% (18)	68.4% (39)
*ycfM*	77.8% (7)	55.2% (16)	78.9% (15)	66.7% (38)

**Table 4:** Temporal distribution of drug sensitivity and resistance phenotype and virulence degree in *Klebsiella pneumoniae* isolates.

	2021 *n* = 9	2022 *n* = 29	2023 *n* = 19	Total (*n* = 57)
Multiresistance phenotype				
MDR	44.4% (4)	62.1% (18)	78.9% (15)	64.9% (37)
Sensitive	55.6% (5)	37.9% (11)	21.1% (4)	35.1% (20)
ESBL producing strains				
Positive	33.3% (3)	65.5% (19)	78.9% (15)	64.9% (37)
Negative	66.7% (6)	111.1% (10)	44.4% (4)	35.1% (20)
Virulence degree				
High *	33.3% (3)	48.3% (14)	73.7% (14)	54.3% (31)
Low *	66.7% (6)	51.7% (15)	26.3% (5)	45.6% (26)

MDR—Multidrug resistance; ESBL—Extended Spectrum Beta Lactamase. * High virulence—between 5 and 8 virulence genes; Low virulence—between 1 and 4 virulence genes.

**Table 5:** Comparison of resistance and virulence-associated gene frequencies between high and low virulence-gene burden *Klebsiella pneumoniae* isolates.

	High Virulence Gene Burden Strains (*n* = 21)	Low Virulence Gene Burden Strains (*n* = 18)	*p* *
*blaCTX-M*	47.6% (10)	16.7% (3)	0.050
*aac(6′)-Ib*	57.1% (12)	16.7% (3)	0.019
*entB*	85.7% (18)	16.7% (3)	<0.001
*ybtS*	57.1% (12)	16.7% (3)	0.019
*fimD*	85.7% (18)	44.4% (8)	0.015
*mrkC*	81.0% (17)	38.9% (7)	0.010
*wabG*	81.0% (17)	11.1% (2)	<0.001
*uge*	85.7% (18)	22.2% (4)	<0.001
*ycfM*	90.5% (19)	38.9% (7)	0.002

High virulence-gene burden was defined as the presence of 5–8 virulence-associated genes, whereas low virulence-gene burden was defined as the presence of 1–4 genes. Comparisons of individual virulence-associated genes were considered descriptive and exploratory because these genes contribute to the definition of the virulence-gene burden categories. * *p* < 0.05.

## Data Availability

The original contributions presented in this study are included in the article. Further inquiries can be directed to the corresponding authors.

## Supplementary Materials

The following supporting information can be downloaded at: https://www.mdpi.com/article/10.3390/children13091165/s1, Table S1: Maternal, Neonatal, Clinical, and Outcome Characteristics by Year.
