# Supplementary material for: Molecular Characterization and Clinical Outcomes of Klebsiella pneumoniae in Neonatal Sepsis: A Three-Year Molecular Epidemiological Study
Source: Children (Basel). 2026 Aug 29;13(9):1165. doi: 10.3390/children13091165 (PMC13605336; doi:10.3390/children13091165)
Supplement: Supplementary file 1 [file children-13-01165-s001.zip › children-4475858-supplementary.pdf]

Table S1. Maternal, Neonatal, Clinical, and Outcome Characteristics by Year

Characteristics+	2021^ n=9, % (n)	2022^ n=18, % (n)	2023^ n=12, % (n)
**Maternal characteristics**			
PROM	33.3% (3)	50.0% (9)	8.3% (1)
PPROM	33.3% (3)	44.4% (8)	0.0% (0)
Chorioamnionitis	0.0% (0)	11.1% (2)	0.0% (0)
Cervicovaginitis	22.2% (2)	11.1% (2)	25.0% (3)
**Neonatal characteristics**			
Gestational Age, (Weeks)*	35.5 [30–36.1]	30 [27–35.37]	32.7 [29.7–37.6]
Birth Weight, (g)*	2,070 [1,045–2,245]	1,020 [830–2,163.8]	1,442.5 [1165.0–2,243.5]
**Birth Weight Classification**			
Normal/Adequate	22.2% (2)	16.7% (3)	33.3% (4)
Low Birth Weight	33.3% (3)	22.2% (4)	25.0% (3)
Very Low Birth Weight	33.3% (3)	11.1% (2)	25.0% (3)
Extremely Low Birth Weight	11.1% (1)	50.0% (9)	16.7% (2)
**Sex**			
Male	66.7% (6)	55.6% (10)	50.0% (6)
Female	33.3% (3)	44.4% (8)	50.0% (6)
Apgar Score at 1 min	6 [4–7]	7 [5–8]	6.5 [5–8]
Apgar Score at 5 min	9 [8–9]	9 [8–9]	8.5 [8–9]
**Clinical Signs**			
Fever	44.4% (4)	72.2% (13)	75.0% (9)
Hypothermia	0.0% (0)	11.1% (2)	0.0% (0)
Tachycardia	33.3% (3)	72.2% (13)	33.3% (4)
Bradycardia	0.0% (0)	5.6% (1)	0.0% (0)
Tachypnea	22.2% (2)	22.2% (4)	16.7% (2)
Bradypnea	0.0% (0)	0.0% (0)	0.0% (0)
Apnea	33.3% (3)	44.4% (8)	50.0% (6)
Respiratory Distress Syndrome	66.7% (6)	61.1% (11)	50.0% (6)
**Hematologic Findings**			
Leukopenia	22.2% (2)	44.4% (8)	25.0% (3)
Leukocytosis	22.2% (2)	27.8% (5)	50.0% (6)
Neutropenia	44.4% (4)	50.0% (9)	33.3% (4)
Neutrophilia	44.4% (4)	55.6% (10)	50.0% (6)
Thrombocytopenia	11.1% (1)	55.6% (10)	50.0% (6)
**Complications**			
Thrombosis	22.2% (2)	0.0% (0)	0.0% (0)
Meningitis	11.1% (1)	11.1% (2)	25.0% (3)
Gastroschisis	0.0% (0)	22.2% (4)	8.3% (1)
Fetal Growth Restriction	22.2% (2)	44.4% (8)	33.3% (4)
Enterocolitis	22.2% (2)	27.8% (5)	50.0% (6)

**Interventions and Outcomes**			
Catheter	77.8% (7)	94.4% (17)	91.7% (11)
Mechanical Ventilation	66.7% (6)	61.1% (11)	75.0% (9)
Total Parenteral Nutrition (TPN)	50.0% (4)	66.7% (12)	91.7% (11)
Length of Hospital Stay, (days)	86 [29–117]	80.5 [65.2–140.5]	79.5 [52.8–105]
Mortality	11.1% (1)	5.6% (1)	8.3% (1)

+ Data correspond to the clinical characteristics of patients at the time of the first *K. pneumoniae* isolation.

^ All cases corresponded to late-onset sepsis (LOS; >72 hours after birth).

* Data are presented as median values with the first and third quartiles (Q1–Q3).
